# Ontario Veterinary College

**Published:** 1887-07

**Authors:** 


### PROCEEDINGS VETERINARY MEDICAL SOCIETIES.

### ONTARIO VETERINARY COLLEGE.

On March 28th, 29th, 30th and 31st, the examination of the students of the Ontario Veterinary College, for the diploma of the Agricultural and Arts Association of Ontario, took place before the official Board of Examiners; the oral and written examinations specially for honor work, having been held during the two weeks previous to the meeting of that Board.

The results of all the examinations, pass and honor, were announced at the closing exercises of the College, Temperance Hall, on the 31st of March.

The spacious lecture room was filled to the doors with students and visitors. Among those present were his Honor, Lieutenant-Governor Robinson, President Wilson of the University of Toronto Mayor Howland, John Leys, M. P. P., Charles Drury, M. P. P., G. R. R. Cockburn, M. P., Fred. Denison, M. P., Rev. G. M: Milligan, Dr. Thorburn, Dr. Duncan, Dr. Coleman of Ottawa, and others. The Lieutenant Governor, and several other gentlemen assisted in the distribution of medals and prizes. Professor Andrew Smith, F. R. C. V. S., President of the College presided. After the reading of the names of the graduates, and the presentation of prizes, Lieutenant-Governor Robinson briefly spoke, eulogizing in the highest terms, the natural ability and requirements of President Smith, and congratulated that gentlemen on the brilliant successes he had achieved in promoting the interests of the Ontario Veterinary College. How the graduates of the College had succeeded in the United States, was shown by the large number of American students who presented themselves at every session. These men had given Canada and her famous Veterinary Institution a good name abroad, and he hoped they would continue to do so.

President Wilson in briefly wishing every success to the Ontario Veterinary College and its able President, stated that, although entirely unconnected with it himself, the wide-spread reputation of the institution was constantly being impressed upon him by the letters which he often received referring to it. He considered that the possession of such a College was of immense advantage to Canada.

Mayor Howland spoke of the pleasure with which he, as Mayor of the City of Toronto, attended on this occasion. He considered that a college with three hundred and fifty students, sending out, as it was doing this year, over one hundred graduates, was a power before which quackery would soon come to an end.

Rev. G. M. Milligan, Col. Denison, M. P., W. Shaw, V. S., of Dayton, Ohio, (President of the Ontario Veterinary Medical Association), Alderman Dodds and others spoke warmly of the College, its Faculty and the graduates.

Alderman Dodds, in the course of his remarks, paid an eloquent tribute to the memory of Dr. Barrett, late Professor of Physiology in the College.

Professor Smith, President of the College, in closing, referred to the remarkable success of the past session. So greatly had the attendance increased of late, that the classes had outgrown the largest expectations of the Faculty. Before the commencement of another session, large new lecture-rooms, laboratories and every convenience for scientific work would be built.

The following is a list of the graduates:

Ernest K. Alexander, Hoosac, N. Y.; Edmund Austin, Simcoe, Ont.; Amos S. Barnes, Fingal, Ontario; Coral D. Beardsley, Union Springs, N. Y.; W. H. Beatty, James B. Black, Brampton, Ont.; John E. Blackwell, London, Ont.; J. C. Booker, Jerseyville, Ill.; James Bowler, Amherstburg, Ont,; Louis R. Brady, Manhattan, Kan.; John E. Browne, Wilmington, Ohio.; John D. Buskirk, Bradley, Mich.; Irwin J. Carter, Toledo, Ohio; W. G. Carmichael, Seaforth, Ont.; Harry D. Chamberlain, Waterloo, Ind.; Charles G. Chase, Consecon, Ont.; Eugene Coffin, Monticello, Ill.; John W. Cook, Clinton, Ont.; Samuel E. Coseford, Hollin, Ont.; James G. Clarke, Little Briton, Ont.; William F. Clarke, Goderich, Ont.; R. L. Craig, Hamilton, Ont.; James Creamer, Belmont Ont.; Marcus H. Crosby, Uxbridge, Ont.; John J. Culp, Orangeville, Ont.; J. M. Curphey, Rochester, Ind.

Hedley H. Davidson, Burlington, Ont.; Walter G. Dodds, Toronto, Ont.; G. B. Du Bois, Nicholson, Pa.; William O. Dyer, Belmont, Ont.

John S. Evans, Chesley, Ont.; Henry A. Fee, Tiverton, Ont.; Gerald J. Fitzgerald, Lucan, Ont.; Benjamin Fletcher, Oxford Mills, Ont.; Arthur G. Foot, Reading, Pa.; Harry W. Fry, Dunville, Ont.; W. J. Gardenier, Quincy Ill.; Joseph R. Grime, Blackpool, Eng.; James I. Gibson, Kemptville, Ont.; William A. Giffen, Mayfield, Ont.; Joseph R. Gillem, Belleville, Ont.; Charles R. Good, Williamsport, Pa.; Michael R. Grainger, Thamesville, Ont.; Joseph H. Goulding, Greenville, Mich.; James G. Harris, Ashburn, Ont.; Campbell S. Harston, Westminster, Md.; Donald Henderson, London, Ont.; Theries D. Hinebauch, Vicksburg, Mich.; Nicolas Hoffman, Buffalo, N. Y.; Ronald G. Holland, Wellington, Ohio; Fred B. Hollister, Montrose, Pa.; Charles E. Hollingsworth, Hillsboro, Ohio; William H. Hopkins, Green River Ont.; Ben Howes, Carlton Station, N. Y.; Ashton Hutchings, Stratford, Ont.; Sidney L. Hunter, Bath, N. Y.; Frank Hunt, Jamestown, N. Y.

Fred M. Jeffry, Tiro, Ohio; David King, Toronto, Ont.; John King, Dresden, Ont.; Thomas A. King, Brampton, Ont.; Benjamin W. Kitely, Sharon, Ont,; Henry C. Klicker, Clarence, Erie Co., N. Y.; John J. Kline, Allentown, Pa.; J. F. Lavery, Uxbridge, Ont.; Thomas W. McDermott, Le Roy, Ohio; Andrew

McFadden, Dunblain, Ont.; Aaron McKenzie, Port Perry, Ont.; Lauchlan McLean, Alliston, Ont.; James L. McMillan, Vernon River, P. E. I.; John C. McMurtry, South March, Ont.; Archie McTaggart, Nassagewaya, Ont.; James McClintock, Galva, Ill.; James D. McVicar, Poplar Hill, Ont.; Geoige E. Morgan, Chicago, Ill.; John Moore, Plainfield, Ont.; Christopher R. Notman, Toronto, Ont.; William Nichols, Uxbridge, Ont.; Samuel Nichols, Coburg, Ont.; Oliver F. Nugent, London, Ont.; Samuel C. Orr, Riley Centre, Kan.; Levi. F. Orr, Canaan, Ohio. John P. Prudham, London, Ont.; Thomas Purvis, Mallorytown, Ont.; Richard E. Reycraft, Muirkirk, Ont.; William H. Richards, Greenville, Ohio; Harry S. Richards, Wooster, Ohio; John C. Rodgers, Ayr, Ont.; Samuel C. Rudd, Guelph, Ont.; John D. Rutherford, Lucknow, Ont.

Alexander W. Shields, Molton, Ont.; James A. Sinclair, Uxbridge, Ont.; James M. Sloan, Vicksonburg, Pa.; John Steen, Harwich, Ont.; S. S. Snider, Lawrenceville, Ohio; Owen W. Snyder, Lynnville, Pa.. James Sullivan, Almonte, Ont.; Frank Sutterby, Batavia, N. Y.; J. Q. Taylor, Marysville, Ohio; Fred. M. Tye Haysville, Ont.; John H. Veith, Waterloo, Ont: R. M. Waldron, Greensburg, Pa.; William B. Wallace, Middletown, Ohio; H. H. Ward, Rochester, Ind.; Joseph Waring, Thornbury, Ont,; John W. Waters, Fingal, Ont.; Walter F. Weese, Platville, Ill.; James Welsh, Manchester, Ont. P. C. Wilkinson, Claremont, N. H.; Charles E. Winner, Quincy, Pa.; Frank A Wiltrout, Litzenberg, Pa.; Thomas W. Watson, Marshalltown, Iowa; T W. Walton, Warren, Ill.; W. C. Young, Bristol Corners, Que.

Prize and Honor List.—Seniors :

*Pathology.*—Silver Medal, J. C. Booker. Second Prize, R. M. Waldron. Third Prize, J. E. Blackwell, C. E. Winner, equal.

*Anatomy.*—Silver Medal, J. E. Blackwell. Second Prize, R. M. Waldron. Third Prize, H. Fee.

*Entozoa.*—First Prize, F. N. Tye.

*Materia Medica.*—First Prize, J. E. Blackwell. Second Prize, E. Coffin. Third Prize, J. J. Klein, R. M. Waldron, equal.

*Physiology.*—Silver Medal, J. Rutherford. Second Prize, J. E. Blackwell. Third Prize, H. Fee.

*Chemistry.*—First Prize, J. Moore. Second Prize, T. D. Hinebauch. Third Prize, J. G, Harris.

*Microscopy.*—First Prize, C. E. Winner, H. W. Fry, J. Rutherford, equal.

*Bacteriology.*—First Prize, B. Johnson, C. E. Winner, equal. Second Prize, J. E. Blackwell, L. R. Brady, D. King, equal. Third Prize, H. D. Chamberlin, W. T. Weese, equal.

*For the Best General Examination.*—Gold Medal given by the Ontario Veterinary Medical Association. Awarded to J. E, Blackwell, London, Ont.

Juniors :

*Anatomy.*—Silver Medal, W. E. Russell. Second Prize, W. Buiger. Third Prize, E. D. McQueen, S. J. Robinson, equal.

*Chemistry.*—First Prize, T. H. Smythe.

*Physiology.*—First Prize, J. J. Coutts. Second Prize, A. G. Wicks.

*Pathology.*—First R. L. Jamieson. Second Prize, C. D. Morris. Third Prize, W. H. Lindsay, J. D. McGregor, W. E. Russell, N. H. Allis, equal.

